# How time pressure disrupts inhibitory control: neural dissociations between interference and response inhibition

**DOI:** 10.1186/s12993-026-00331-3

**Published:** 2026-04-16

**Authors:** Zitong Ding, Weiwen Wu, Yu Tian

**Affiliations:** 1 Institute of Brain and Psychological Sciences, Sichuan Normal University, 610066 Chengdu, China; 2 Chengdu Swan Lake Primary School, 610101 Chengdu, China

## Abstract

**Background:**

In modern fast-paced societies, subjective time pressure has become a common psychological stressor that may impair inhibitory control, a core component of executive function. Inhibitory control consists of two subprocesses—interference control and response inhibition—yet how time pressure differentially affects these processes and their neural mechanisms remains insufficiently understood.

**Methods:**

This study manipulated subjective time pressure and employed the Flanker task (interference control) and the Go/NoGo task (response inhibition) to examine behavioral performance, event-related potentials (ERPs), and time–frequency oscillations. Forty healthy adults completed both time-pressure and no time pressure conditions. ERP analyses focused on the P1, N1, P2, N2, and P3 components, while time–frequency analyses were performed both across the full frequency range (3–30 Hz) and within specific frequency bands (θ and β), using cluster-based permutation tests.

**Results:**

Behaviorally, time pressure reduced accuracy, especially in incongruent Flanker trials and NoGo trials, while accelerating overall reaction times. ERP results showed enhanced N2 and P3 amplitudes in the Flanker task. In the Go/NoGo task, time pressure led to reduced P2, but increased N2 and P3. Time–frequency analysis revealed higher theta power during early control and increased beta power during later inhibition under time pressure.

**Conclusion:**

Taken together, the behavioral, ERP, and oscillatory findings suggest that subjective time pressure is associated with alterations in inhibitory control, with distinct patterns observed for interference control and response inhibition. Specifically, interference control under time pressure was characterized by enhanced late-stage neural activity without corresponding oscillatory changes, whereas response inhibition was accompanied by modulations in both early and late neural processes, involving enhanced N2 and P3 components as well as increased θ and β band activity. These findings suggest that time pressure may affect interference control and response inhibition through partially distinct neurocognitive mechanisms, rather than a single unified pathway.

**Supplementary Information:**

The online version contains supplementary material available at 10.1186/s12993-026-00331-3.

## Introduction

In contemporary society—defined by escalating competition and increasingly rapid rhythms of daily life—time pressure has become a pervasive stressor affecting students, professionals, and the broader public. Far from being a transient inconvenience, perceived time insufficiency is associated with heightened psychological strain, which contributes to depression [1], diminished well-being [2], and cognitive decline [3, 4]. Understanding how time pressure shapes cognitive processing is therefore crucial for promoting individual health and adaptability in fast-paced environments. Time pressure, defined as the cognitive and emotional strain stemming from the perception that available time is insufficient for task completion [5, 6], has been shown to impair cognitive functioning by depleting cognitive resources, disrupting attentional allocation, and compromising decision-making quality [3, 4, 7]. Recent research further indicates that these detrimental effects of time pressure extend to executive functions as well, particularly in domains involving inhibitory control [8].

As a core component of executive functioning, inhibitory control enables individuals to regulate attention, behavior, and emotion by overriding prepotent internal tendencies or external distractions [9–12]. Research has shown that time pressure can undermine this ability, leading to deteriorated behavioral performance on tasks that require effective response inhibition [13, 14]. According to Nigg’s influential taxonomy, inhibitory control comprises two related yet dissociable subprocesses: interference control—the ability to resist distraction from competing stimuli—and response inhibition—the suppression of a prepotent motor response [15]. These subprocesses are typically measured using the Flanker task [16, 17] and the Go/NoGo task [18, 19], respectively.

The neural activity patterns observed in functional magnetic resonance imaging (fMRI) and electroencephalography (EEG) studies could be interpreted as hinting at a functional dissociation between these two forms of inhibition. fMRI studies show that although both types of inhibition engage a broad network involved in cognitive control, response inhibition tends to rely more on regions that support stopping or overriding actions, such as areas in the lateral prefrontal cortex and parietal cortex. In contrast, interference suppression relies more strongly on regions involved in detecting and resolving conflict, particularly the dorsal anterior cingulate cortex [20]. Complementary ERP research also indicates different timing and scalp distributions for the two subprocesses. Response inhibition typically produces an earlier, more frontally distributed N2 component, reflecting rapid stopping demands, whereas interference suppression is associated with a later, more centrally distributed N2, reflecting conflict monitoring [21]. Together, these findings suggest that the two forms of inhibition involve related but distinct neural processes, which makes it reasonable to expect that time pressure may modulate them differently.

Event-related potentials (ERPs) are voltage fluctuations extracted from the ongoing EEG that are time-locked to sensory, cognitive, or motor events. The time-domain ERP analysis method assumes that the electrical activity elicited by each event is similar, while spontaneous noise is random. This method uses the moment of each event’s occurrence as the standard temporal reference point for data analysis. The EEG signals from each trial are aligned to this standard time point and then averaged. During this averaging process, the noise across different trials cancels out. The resulting averaged EEG signal represents the event-evoked brain activity, reflecting the cognitive processing of the event by the brain [22, 23]. Based on their latency and functional significance, ERPs are commonly divided into two categories: early, sensory-driven (exogenous) components, which peak within approximately 100 ms and depend largely on physical stimulus parameters, and late, cognitively mediated (endogenous) components, which reflect higher-order information processing [24]. Given this methodological foundation, ERPs are ideally suited to trace the rapid dynamics of neural processing due to their millisecond-level temporal resolution, making them a powerful tool for investigating how time pressure modulates distinct stages of cognitive control. In line with this framework, the present study focuses on specific ERP components associated with inhibitory control that map onto distinct processing stages: early components including P1, N1 [25–27], and P2 [28, 29], which index initial sensory and attentional processing within the first 200 ms, and later components—most prominently the N2 and P3—which are known to reflect inhibitory control processes. The N2, a frontocentral negativity emerging 200–400 ms post-stimulus, is associated with conflict monitoring, response inhibition, and interference control [30–35]. The P3, occurring 300–700 ms, reflects the evaluation of inhibition outcomes and the allocation of cognitive resources [33, 36–38]. Increased P3 amplitude is generally interpreted as reflecting a greater allocation of cognitive resources [39–42].

Based on prior research, interference control and response inhibition do not differ in the early P1/N1 components, which reflect stimulus-driven perceptual encoding. Their divergence emerges at the P2 stage: response inhibition depends on early sensory gating, such that failures in this process—indexed by increased P2 amplitude—allow irrelevant stimulus features (e.g., spatial location) to be further processed and ultimately bias responding, whereas errors in interference control do not show such P2 modulation [43]. This pattern supports the view that a reduced P2 amplitude reflects successful early filtering of irrelevant sensory information [44]. Within this theoretical framework, we propose that time pressure elicits task-specific early processing strategies. In the Go/NoGo task, participants may enhance early suppression of task-irrelevant features to cope with time constraints, leading to a decrease in P2 amplitude. In contrast, in the Flanker task, where distractors are essential for conflict detection and cannot be filtered out, early sensory gating is not applicable; thus, no significant P2 differences are expected.

While P2 reflects early sensory gating, later stages of control may be affected more broadly by time pressure. According to Hockey’s compensatory control framework, when time constraints elevate task demands beyond available processing capacity, individuals mobilize additional cognitive resources to maintain task performance [45]. This compensatory upregulation is expected to amplify neural markers of conflict detection and top-down regulation. Consequently, under time pressure, both N2 and P3 amplitudes should increase, reflecting heightened engagement of cognitive control mechanisms.

In EEG research, ERPs capture only phase-locked ‘evoked’ activity through signal averaging, which treats non-phase-locked ‘induced’ oscillations as noise. Time-frequency analysis methods, such as wavelet analysis, address this limitation. It employs finite, rapidly attenuating wavelets (e.g., the complex Morlet wavelet commonly used in EEG). A family of wavelets is generated by scaling (compressing/dilating) a mother wavelet, which are then convolved with the EEG signal. This process provides an adaptive time-frequency resolution—high frequency resolution at low frequencies and high temporal resolution at high frequencies. Time–frequency analysis quantifies neural oscillatory power across time, enabling a more complete characterization of spectral dynamics beyond phase-locked ERPs [46]. Among these oscillations, theta (θ)-band activity (4–7 Hz) plays a central role in inhibitory control. It reliably increases during conflict monitoring and cognitive control demands [47, 48]. θ power is particularly sensitive to task-induced interference: it shows the greatest enhancement in NoGo tasks, followed by the Flanker task, and the smallest effects in the Simon task, reflecting variations in conflict processing demands across paradigms [49]. In contrast, beta (β)-band activity (13–30 Hz) is strongly linked to motor inhibition and the suppression of action tendencies. For example, in the Stop-Signal Task, successful stopping elicits stronger β power than both response trials and failed stops [50–52]. Changes in these frequency bands can thus elucidate how time pressure modulates inhibitory control at the neural level.

While substantial progress has been made in understanding time pressure’s effects on inhibitory control, critical gaps remain in characterizing how it differentially impacts distinct inhibitory processes—particularly the dissociation between interference control and response inhibition. Existing research has predominantly employed objective time manipulations [53]. Though such approaches provide precise experimental control, they may lack ecological validity as real-world time pressure often arises from subjective perceptions rather than actual time constraints, such as when handling complex tasks or multitasking despite having sufficient time. In contrast, subjective time pressure manipulations [7, 54, 55] better capture these psychological experiences, as evidenced by findings that subjective pressure can independently impair performance even without objective time limits [56]. Behavioral measures provide the foundation for understanding brain activity, while EEG techniques—particularly ERPs and time–frequency analyses—serve as a complementary approach, revealing the temporal and spectral dynamics underlying cognitive control. ERPs offer millisecond-level resolution of distinct processing stages, whereas time–frequency analyses capture oscillatory mechanisms associated with inhibitory control. By integrating behavioral, ERP, and time–frequency measures, our study offers a more comprehensive characterization of how subjective time pressure modulates interference control and response inhibition.

The present study aims to bridge these gaps by integrating behavioral, ERP, and oscillatory analyses to investigate the effects of subjective time pressure on inhibitory control. Using the Flanker task (interference control) and the Go/NoGo task (response inhibition), we manipulate time pressure subjectively through specific instructions. We focus on examining five key ERP components (P1, N1, P2, N2, P3) and two frequency bands (θ, β) that are known to be associated with cognitive control processes. Our hypotheses are as follows:

1. Time pressure will specifically impair the cognitive process of interference control during the Flanker task. This impairment will be reflected in deteriorated behavioral performance. At the ERP level, it will be reflected in increased N2 and P3 amplitudes. Furthermore, time pressure will modulate neural oscillations, leading to increased θ band power in later processing stages. In contrast, the early sensory processing components (P1 and N1) and the P2 component are expected to remain unaffected.
2. Similarly, time pressure is expected to compromise response inhibition in the Go/NoGo task, resulting in decreased behavioral performance. At the ERP level, this effect will manifest as a reduction in P2 amplitude followed by increases in N2 and P3 amplitudes. In the time–frequency domain, these ERP dynamics will be accompanied by increased θ and β power. In contrast, the early sensory processing components (P1 and N1) component are expected to remain unaffected.

## Methods

### Participants

Behavioral and EEG data were collected from 50 healthy adults (18–25 years old). All participants reported normal or corrected-to-normal vision and had no history of neurological or psychiatric disorders, as verified through self-report and a brief clinical interview. Prior to the experiment, participants completed the Depression Anxiety Stress Scales-21 (DASS-21) [57] and the Beck Depression Inventory-II (BDI-II) [58] to screen for depressive symptoms and anxiety levels.

Participants with BDI-II scores exceeding 17 (indicating clinical depression) or DASS-21 anxiety subscale scores exceeding 14 (indicating high anxiety) were excluded. Additionally, participants with more than 25% of trials contaminated by EEG artifacts (e.g., ocular movements, muscle activity) in either task were excluded. To ensure cross-task comparability, only those with valid EEG data for both Flanker and Go/NoGo tasks were retained. This resulted in a final sample of 40 participants (16 males, 24 females; age: M ± SD = 20.0 ± 2.11 years). The mean BDI-II score in the final sample was 7.15 (SD = 4.22), and the mean DASS-21 anxiety subscale score was 7.85 (SD = 5.12).

All participants provided written informed consent prior to the experiment and received course credit or monetary compensation for their participation. The study procedures adhered strictly to the ethical standards outlined in the Declaration of Helsinki (World Medical Association, 2013) and were approved by the local institutional review board of (IRB No. SCNU-2410311).

### Procedure

Participants completed the experimental tasks while seated comfortably in a dimly lit, sound-attenuated room. The experiment consisted of two phases: a practice phase and a formal testing phase.

Practice Phase: During the practice phase, participants performed 80 trials of the Flanker task (40 congruent, 40 incongruent) and 20 trials of the Go/NoGo task (14 Go trials, 6 NoGo trials) to familiarize themselves with the task demands. EEG electrodes were applied after the practice phase to minimize participant discomfort.

Formal Experiment: The formal experiment employed a 2 (Time Pressure: present vs. absent) × 2 (Task Type: Flanker vs. Go/NoGo) within-subjects design, with all participants completing all four experimental conditions. To counterbalance potential order effects, participants were randomly assigned to one of four task sequence combinations (e.g., Flanker-Time Pressure first, Go/NoGo-No Time Pressure first, etc.). The entire session lasted approximately 60 min.

Time Pressure Manipulation: Time pressure was manipulated using instructional prompts. In the time pressure condition, participants were told: “In this phase, the time to complete the task is limited. Once you start, the timer will begin, and breaks are not counted. You must finish within the expected time; otherwise, it may affect the results.” In fact, no objective time limit was set; the instructions were intended to induce a subjective sense of pressure and urgency. In the no time pressure condition, participants were told: “Please follow the task instructions carefully.” To assess the effectiveness of the manipulation, participants rated their perceived time pressure on a 0–100 visual analog scale (0 = none, 50 = moderate, 100 = extreme) at the end of each task block. Additionally, they completed the Time Pressure Questionnaire (TPQ) [59], a validated measure of subjective time pressure.

**Fig. 1:**
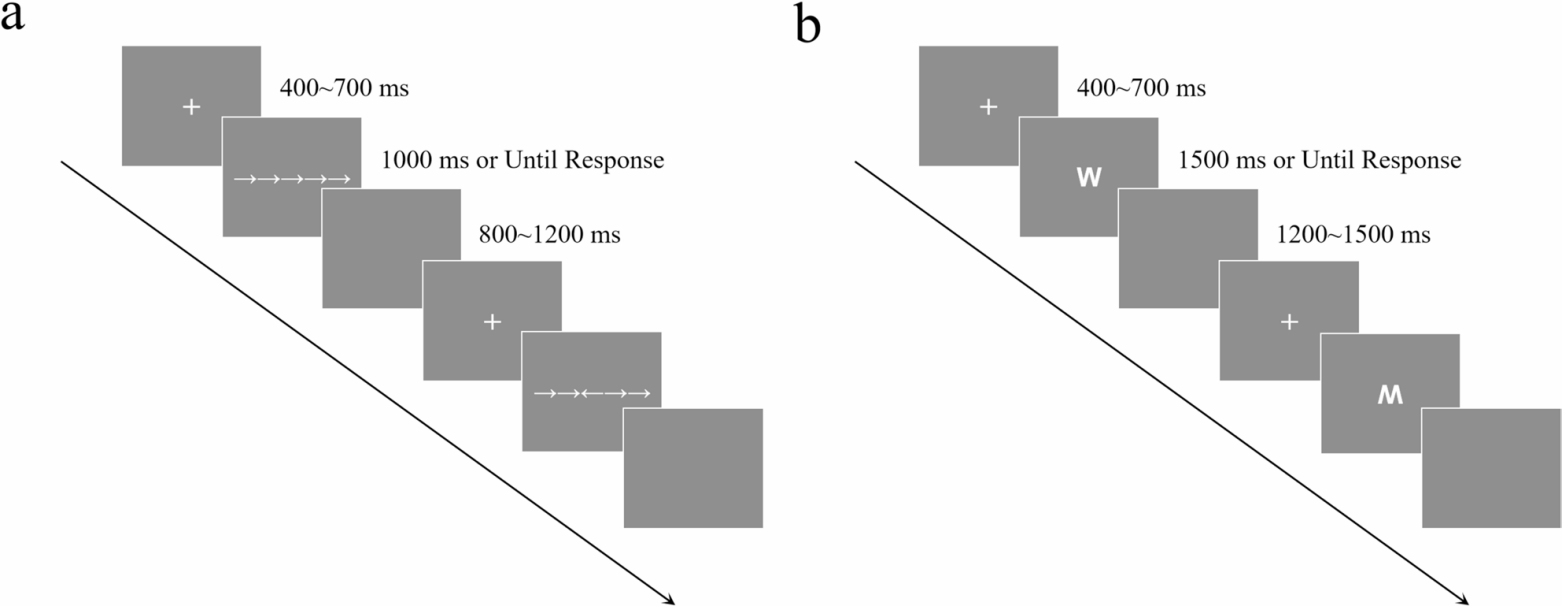
Schematic illustration of the experimental procedures for the two task conditions. **a** The Flanker Task sequence. **b** The Go/NoGo Task sequence

The Flanker task was used to assess interference control. The task consisted of 240 trials (120 congruent, 120 incongruent), with stimuli presented in a randomized order. In congruent trials, the central arrow aligned with the flanking arrows (e.g., “→→→→→”, “←←←←←”); in incongruent trials, the central arrow conflicted with the flankers (e.g., “→→←→→”, “←←→←←”). Participants were instructed to respond as quickly and accurately as possible to the direction of the central arrow by pressing the “F” key (left) or “J” key (right). Each trial began with a fixation cross (“+”) displayed for 400–700 ms (jittered), followed by the stimulus (1000 ms maximum duration or until response). A blank screen (800–1200 ms jittered) separated trials (see Fig. 1a).

The Go/NoGo task was used to assess response inhibition. The task consisted of three blocks, each with 120 trials (84 Go trials, 36 NoGo trials). In Go trials, participants pressed the “G” key in response to the inverted letter “W” (presented at the center of the screen). In NoGo trials, they withheld a response to the upright “W”. Stimuli were presented in a pseudorandom sequence to ensure that NoGo trials did not occur more than three times consecutively. Each trial began with a fixation cross (“+”) displayed for 400–700 ms jittered, followed by the stimulus (1500 ms maximum duration or until response). A blank screen (1200–1500 ms jittered) separated trials (see Fig. 1b). 

### EEG data recording and preprocessing

EEG signals were recorded using a 64–channel ActiCap system (Brain Products GmbH, Germany) with electrodes positioned according to the extended international 10–20 system. FCz served as the online reference electrode, AFz as the ground electrode, and electrodes below the right eye recorded vertical electrooculogram (VEOG) to monitor eye movements and blinks. Scalp impedance at each electrode site was maintained below 5 kΩ throughout recording. EEG data were sampled at 1000 Hz with a hardware low-pass filter of 250 Hz.

Offline Preprocessing: EEG data were processed offline in MATLAB using the EEGLAB toolbox [60]. All EEG signals were re-referenced to the average of the mastoids (M1 and M2). A band-pass filter (0.1–30 Hz, zero-phase shift, 12 dB/octave roll-off) and a notch filter at 50 Hz were applied to remove environmental noise and power line artifacts. Bad channels were identified via visual inspection and the pop_rejchan function and were subsequently interpolated using spherical spline interpolation. Noisy segments were manually removed after visual inspection of the continuous EEG trace. Independent Component Analysis (ICA) was performed to remove ocular artifacts (eye blinks and movements), with artifact components identified using the ADJUST plugin. After artifact correction, EEG data were epoched from − 600 ms to 800 ms relative to stimulus onset, and baseline correction was applied using the pre-stimulus period. Epochs exceeding ± 80 µV were rejected.

ERP Analysis: For the time-domain ERP analysis, epochs from − 200 ms to 800 ms relative to stimulus onset were extracted from the preprocessed EEG data. Each epoch was baseline-corrected using the − 200 to 0 ms interval. ERPs were computed separately for each condition by averaging across all artifact-free trials. For each ERP component (P1, N1, P2, N2, P3), the temporal windows and electrode sites were defined a priori based on established ERP literature employing Flanker and Go/NoGo paradigms, which delineates the typical latencies and canonical scalp regions where these components reach maximal amplitudes. These theory-driven specifications formed the basis for subsequent analyses. Specifically:

P1: 80–130 ms at Oz, O1, O2 (occipital region).

N1: 130–180 ms at Fz, FCz, Pz, Oz (fronto-central and occipital regions).

P2: 180–250 ms at Fz, FCz, Cz (fronto-central region).

N2: 200–400 ms at Fz, FCz, Cz (fronto-central region).

P3: 300–700 ms at Cz, CPz, Pz (centro-parietal region).

Time–Frequency Analysis: Time–frequency decomposition was performed using the FieldTrip toolbox [61]. For each participant, preprocessed EEG data were first converted from EEGLAB structures into FieldTrip format. Time–frequency representations (TFRs) of oscillatory power were computed using a Morlet wavelet transform. Following recommendations regarding wavelet kernel parameters [46], a frequency-dependent wavelet width was adopted to balance temporal and spectral resolution across the analyzed spectrum. Specifically, wavelet widths increased linearly from 3 to 7 cycles across the 3–30 Hz frequency range, providing higher temporal precision at lower frequencies (e.g., θ band) and improved frequency resolution at higher frequencies (e.g., β band). The analysis window spanned − 0.6 to 0.8 s relative to stimulus onset, with a temporal resolution of 2 ms.

Following wavelet decomposition, power spectra were averaged across trials for each condition. Baseline correction was performed using a decibel (dB) transformation relative to the − 0.5 to − 0.2 s prestimulus interval for each frequency band, thereby normalizing power fluctuations across participants and conditions. Event-related spectral perturbations (ERSPs) were subsequently computed for each condition.

### Behavioral and EEG data analysis

Behavioral Data Analysis: Behavioral performance was analyzed using a 2 (Time Pressure: No Time Pressure vs. Time Pressure) × 2 (Trial Type: Congruent vs. Incongruent for the Flanker task; Go vs. NoGo for the Go/NoGo task) repeated-measures analysis of variance (ANOVA). Reaction times (RTs) and accuracy rates were analyzed separately. For the RT analysis, only trials with correct responses were included. RT preprocessing was conducted separately for the Flanker and Go/NoGo tasks. For each task, all correct RTs across its conditions were pooled to compute a task-specific overall mean and standard deviation. Trials with RTs shorter than 100 ms or exceeding ±3 standard deviations from this mean were removed. Condition-wise mean RTs were then calculated within each task based on the remaining trials. Post hoc pairwise comparisons were Bonferroni-corrected, with statistical significance set at P < 0.05. EEG Data Analysis: EEG data were analyzed to examine the effects of time pressure on neural activity, including ERP and time–frequency analyses.

For the ERP analysis, difference waves were computed for each task (Incongruent–Congruent for the Flanker task; NoGo–Go for the Go/NoGo task). Based on the scalp topographies of each component and prior findings regarding their canonical spatial distributions, the electrode sites were preliminarily determined. A repeated-measures ANOVA with factors Time Pressure (No Time Pressure vs. Time Pressure) and Electrode Site was then performed on the difference waves to determine the electrode exhibiting the largest component-specific amplitude differences, which was subsequently selected as the representative site for statistical analysis. The temporal windows for each ERP component were determined based on well-established literature and further informed by the characteristic morphology of the grand-average waveforms in the present dataset. Mean amplitudes of the component-specific difference waves were then extracted from the representative electrode within these defined time windows. Paired-sample t-tests were conducted on the extracted mean amplitudes to evaluate the effects of time pressure on component-level neural activity. Statistical significance was set at *P* < 0.05.

For the time–frequency analysis, difference power was first computed for each task (Incongruent–Congruent for the Flanker task; NoGo–Go for the Go/NoGo task). Electrode sites were selected a priori based on the ERP results to ensure consistency between time-domain and time–frequency analyses.

Cluster-based permutation tests were then conducted using a paired-samples t statistic to directly compare the Time Pressure (TP) and No Time Pressure (NTP) conditions at the sensor level. The analysis covered the full post-stimulus interval (0–0.8 s) and the full frequency range of interest (3–30 Hz), while being restricted to the predefined electrode sites. A cluster-forming threshold of α = 0.05 and 5000 Monte Carlo permutations were applied. This approach enabled the identification of spatiotemporal–frequency clusters showing significant TP–NTP differences, while appropriately controlling for multiple comparisons across time, frequency, and sensors.

To facilitate functional interpretation, additional hypothesis-driven analyses were conducted within the θ (4–7 Hz) and β (13–30 Hz) frequency bands. For each significant cluster, mean oscillatory power was extracted for each participant by averaging across the electrodes, time points, and frequency bins that constituted the cluster. These cluster-wise mean power values were used for visualization and subsequent correlational analyses.

## Results

### Manipulation check for time pressure

**Fig. 2:**
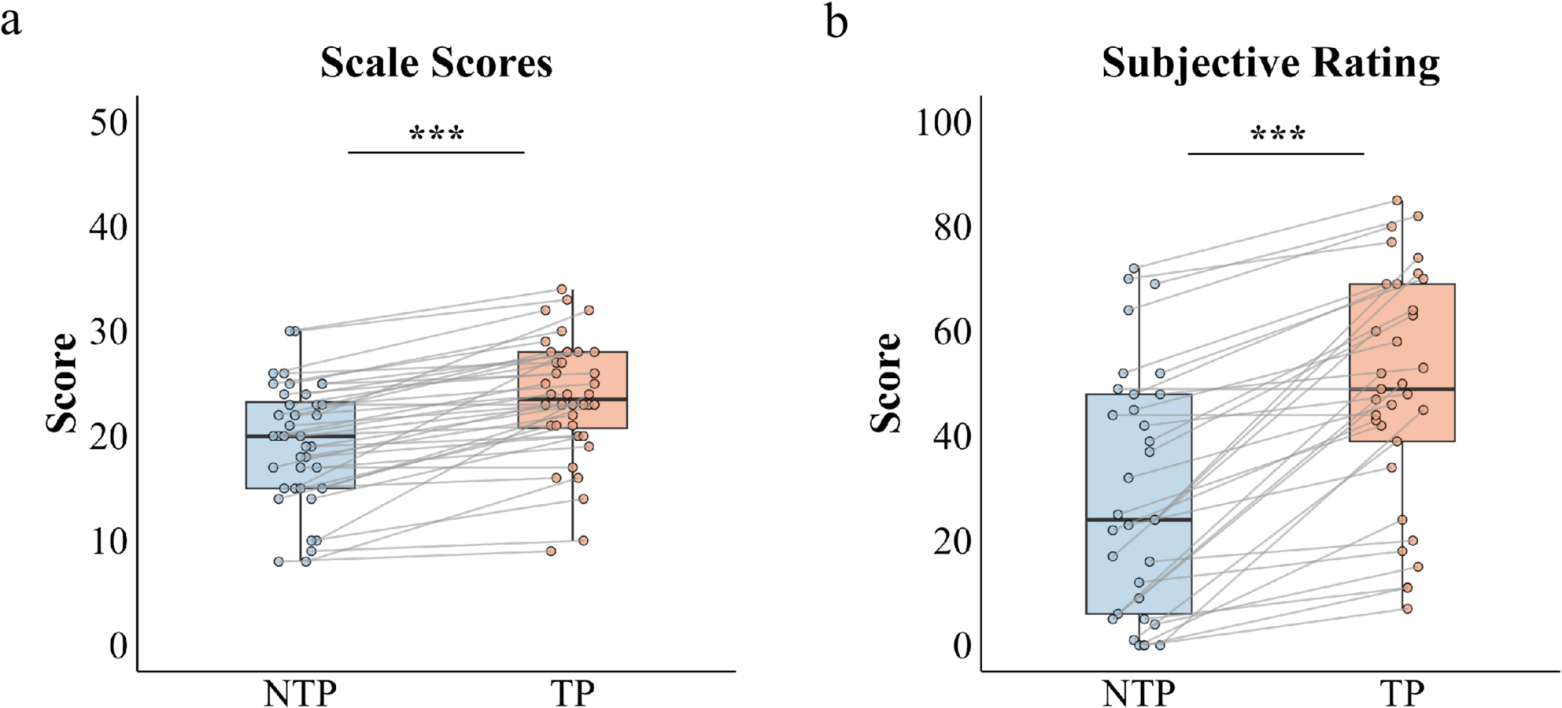
Verification of the time pressure manipulation. **a** Boxplots of average on the time pressure scale under time pressure (TP) and no time pressure (NTP) conditions. **b** Boxplots of average subjective ratings of perceived time pressure under time pressure (TP) and no time pressure (NTP) conditions. Individual data points (dots) represent values for each participant. Gray lines connect paired observations from the same participant across conditions. Asterisks indicate statistical significance: ^*^P < 0.05, ^**^P < 0.01, ^***^P < 0.001

To verify the effectiveness of the time pressure manipulation, paired-sample t-tests were conducted on time pressure scale scores and subjective ratings under time pressure and no time pressure conditions. The results indicated that participants reported significantly higher time pressure scale scores in the time pressure condition (M =23.55, SD = 5.724) than in the no time pressure condition (M = 19.40 , SD = 5.665 ), *t*(39) = 8.144 , *P* < 0.001, 95% CI = (0.862, 1.704), Cohen’s *d* = 1.288 (see Fig. 2a). Similarly, subjective ratings of time pressure were significantly higher in the time pressure condition (M = 46.15, SD = 25.505) than in the no time pressure condition (M = 29.45, SD = 25.179), *t*(39) = 6.709, *P* < 0.001, 95% CI = (0.668, 1.445), Cohen’s *d* = 1.061. These findings confirm that the time pressure manipulation was effective (see Fig. 2b).

### Flanker task results

**Fig. 3:**
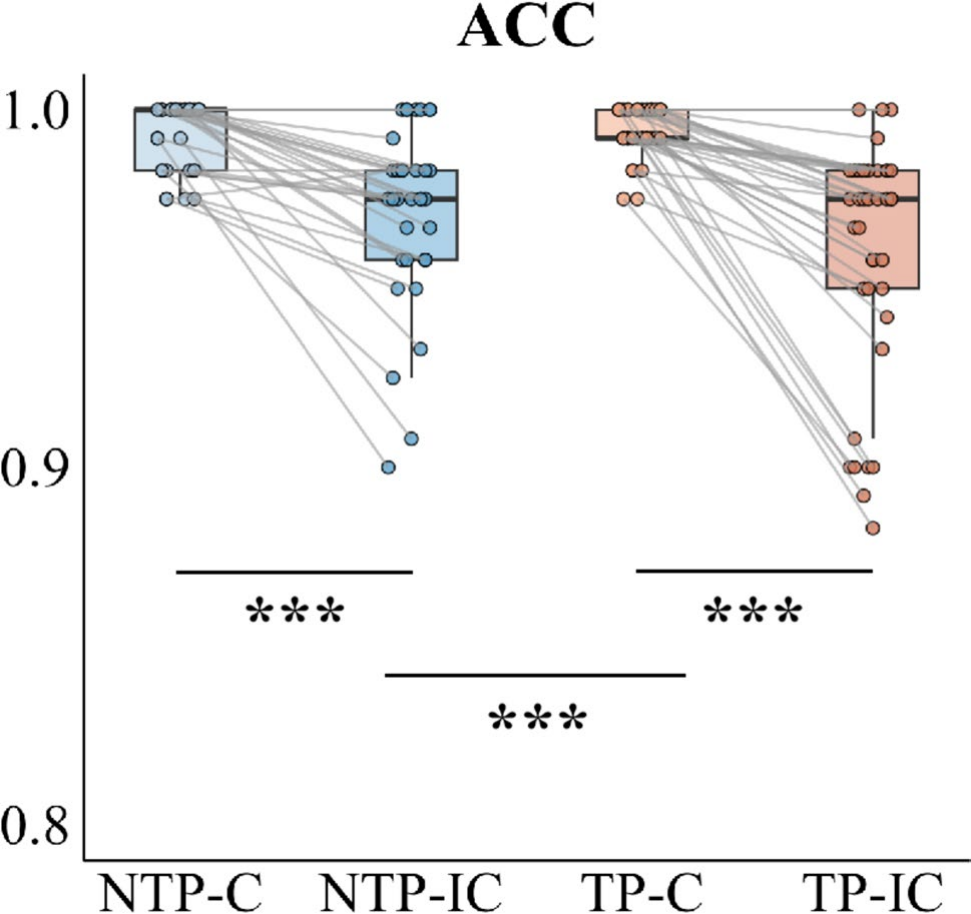
Behavioral performance in the Flanker task under time pressure and no time pressure conditions. **a** Boxplots of average accuracy rates for congruent (C) and incongruent (IC) trials under time pressure and no time pressure conditions. Individual data points (dots) represent values for each participant. Gray lines connect paired observations from the same participant across conditions. Asterisks indicate statistical significance: ^*^P < 0.05, ^**^P < 0.01, ^***^P < 0.001

Behavioral Performance: A two-way repeated measures ANOVA was conducted on accuracy rates in the Flanker task, with time pressure and trial type as within-subjects factors. The interaction between time pressure and trial type was significant, *F*(1, 39) = 13.651, *P* < 0.001, *η*_p_² = 0.259. Simple effects analysis showed that while time pressure had no significant effect on congruent trials, *F*(1, 39) = 0.789, *P* = 0.380, *η*_p_² = 0.020, it significantly reduced accuracy for incongruent trials, *F*(1, 39) = 9.470, *P* = 0.004, *η*_p_² = 0.195. Furthermore, the typical congruency effect (higher accuracy for congruent vs. incongruent trials) was observed in both time pressure, *F*(1, 39) = 40.389, *P* < 0.001, *η*_p_² = 0.509, and no time pressure conditions, *F*(1, 39) = 20.724, *P* < 0.001, *η*_p_² = 0.347, with the effect being more pronounced under time pressure, Under time pressure, accuracy was significantly lower for incongruent trials (M = 0.958, SD = 0.038) compared to congruent trials (M = 0.994, SD = 0.007), whereas in the no time pressure condition, incongruent trials showed moderately reduced accuracy (M = 0.971, SD = 0.030) relative to congruent trials (M = 0.992, SD = 0.010). The analysis also revealed significant main effects of both time pressure, *F*(1, 39) = 4.977, *P* = 0.032, *η*_p_² = 0.113, and trial type, *F*(1, 39) = 36.235, *P* < 0.001, *η*_p_² = 0.482. Participants demonstrated lower accuracy under time pressure (M = 0.976, SD = 0.019) compared to no time pressure conditions (M = 0.982, SD = 0.019), and lower accuracy for incongruent trials (M = 0.964, SD = 0.032) relative to congruent trials (M = 0.993, SD = 0.006; see Fig. 3).

The two-way repeated measures ANOVA for reaction times (RTs) revealed no significant interaction between time pressure and trial type, *F*(1, 39) = 0.045, *P* = 0.834, *η*_p_² = 0.001, indicating that the effect of time pressure on RTs did not differ between congruent and incongruent trials. Significant main effects were observed for both factors. The main effect of time pressure was significant, *F*(1, 39) = 9.55, *P* = 0.004, *η*_p_² = 0.197, with participants responding faster under time pressure (M = 458.0 ms, SD = 39.535 ms) than under no time pressure conditions (M = 480.072 ms, SD = 52.361 ms). Similarly, the main effect of trial type was significant, *F*(1, 39) = 68.819, *P* < 0.001, *η*_p_² = 0.638, showing faster responses for congruent trials (M = 449.908 ms, SD = 39.674 ms) compared to incongruent trials (M = 488.163 ms, SD = 46.207 ms). 

**Fig. 4:**
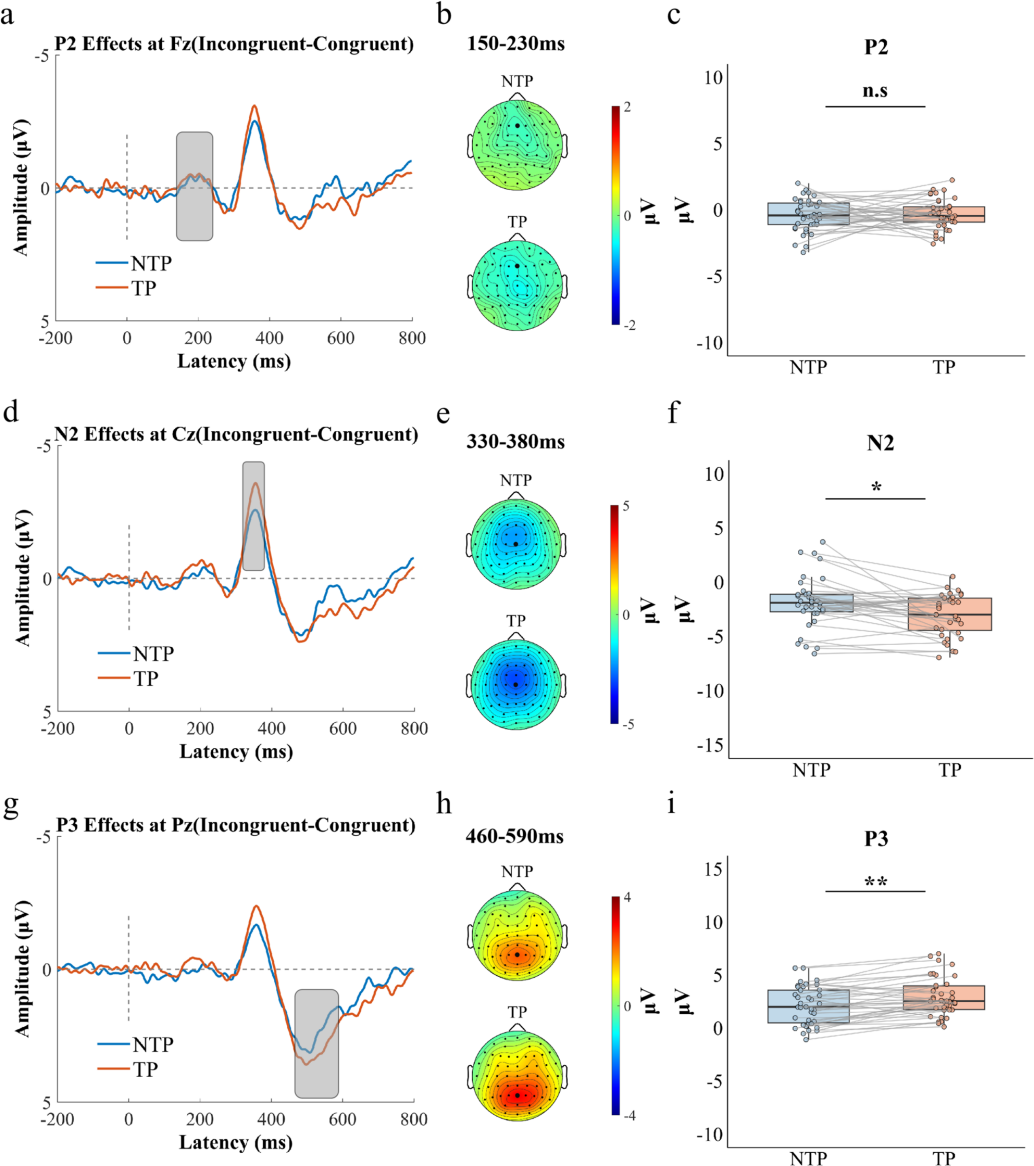
Time pressure effects on P2, N2, and P3 components during interference inhibition. **a** Grand-average ERP waveforms at Fz showing incongruent-congruent difference waves for no time pressure and time-pressure conditions. **b** The voltage topographies show scalp distributions of the maximum differential P2 (150–230ms). **c** Boxplots of P2 amplitude differences under no time pressure and time pressure conditions. **d** Grand-average ERP waveforms at Cz showing incongruent-congruent difference waves for no time pressure and time-pressure conditions. **e** The voltage topographies show scalp distributions of the maximum differential N2 (330–380ms). **f** Boxplots of N2 amplitude differences under no time pressure and time pressure conditions. **g** Grand-average ERP waveforms at Pz showing incongruent-congruent difference waves for no time pressure and time-pressure conditions. **h** The voltage topographies show scalp distributions of the maximum differential P3 (460–590ms). **i** Boxplots of P3 amplitude differences under no time pressure and time pressure conditions. Individual data points (dots) represent values for each participant. Gray lines connect paired observations from the same participant across conditions. Asterisks indicate statistical significance: ^*^P < 0.05, ^**^P < 0.01, ^***^P < 0.001

ERP Results: Paired-sample *t*-tests were conducted to compare difference wave amplitudes (P1, N1, P2, N2, P3) between time pressure and no time pressure conditions. No significant differences were found between conditions for the P1 amplitude at Oz (80–100 ms), the N1 amplitude at Fz (120–150 ms), and the P2 amplitude at Fz (150–230 ms) (all *P*s > 0.05). Significant differences emerged for later components: The N2 difference wave amplitude at Cz (330–380 ms) was significantly more negative under time pressure (M = -2.993 µV, SD = 2.307 µV) compared to no time pressure (M = -2.179 µV, SD = 2.794 µV), *t*(39) = 2.135, *P* = 0.039, 95% CI = (0.017, 0.654), Cohen’s *d* = 0.338. Similarly, the P3 difference wave amplitude at Pz (460–590 ms) was significantly greater under time pressure (M = 3.034 µV, SD = 1.875 µV) than under no time pressure (M = 2.383 µV, SD = 2.104 µV), *t*(39) = 2.936, *P* = 0.006, 95% CI = (0.135, 0.788), Cohen’s *d* = 0.464 (Fig. 4).

**Fig. 5:**
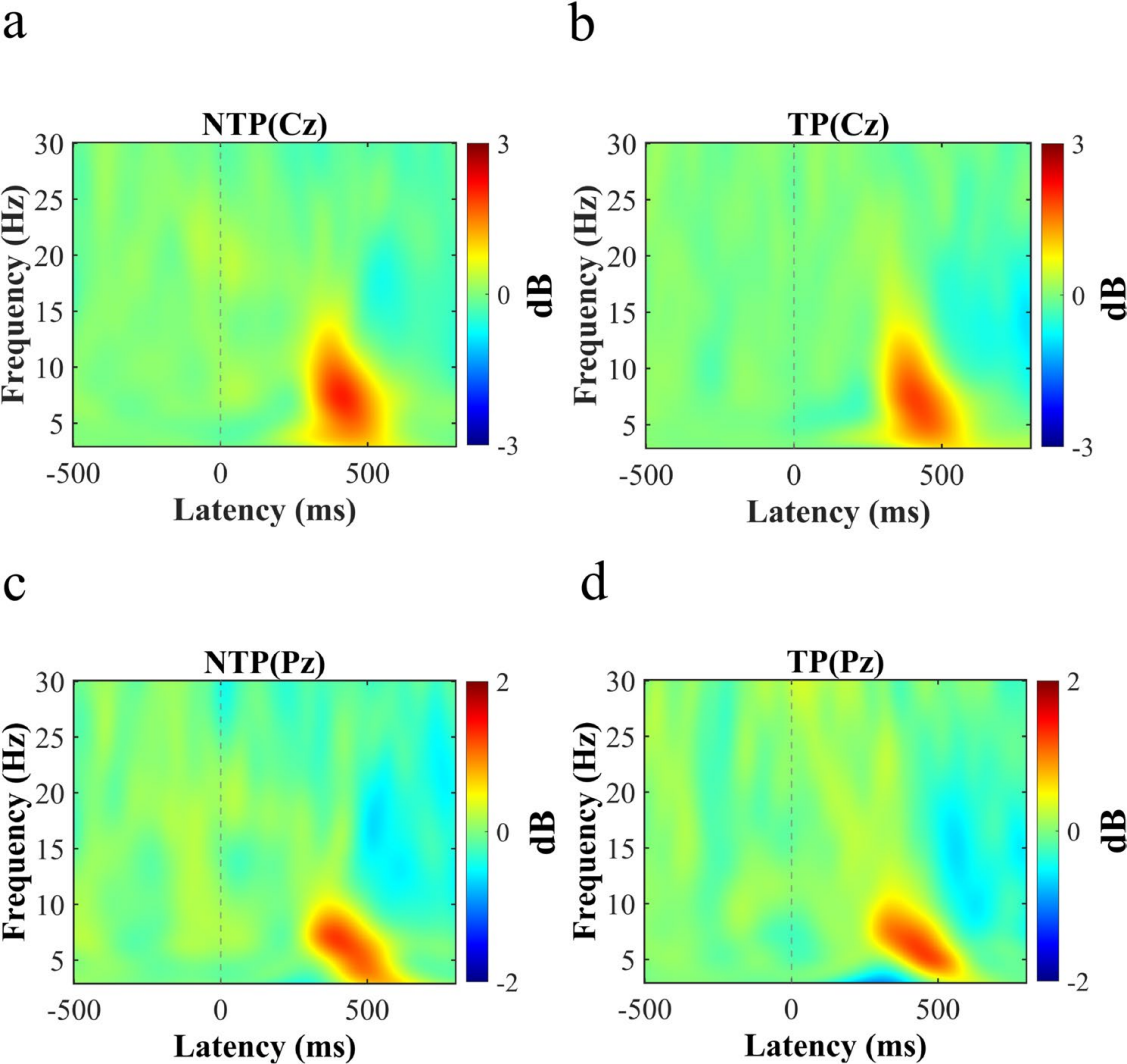
Time-frequency distributions at exemplar electrode sites (Cz and Pz) contrasting conditions without versus with time pressure (Incongruent–Congruent difference). **a**, **b** Power differences at the Cz electrode. **c**, **d** Power differences at the Pz electrode. No significant differences were observed

Time–Frequency Results: For the Flanker task, time–frequency analyses were conducted at the same electrode sites as those used in the time-domain ERP analyses. Cluster-based permutation tests using a paired-samples t statistic were applied to the difference power (Incongruent–Congruent) to examine the effects of time pressure (No Time Pressure vs. Time Pressure) across the full time (0–0.8 s) and frequency (3–30 Hz) ranges. No significant spatiotemporal–frequency clusters were identified in the full-range analysis (all *P*s > 0.05). To facilitate functional interpretation, additional hypothesis-driven analyses were performed within the θ (4–7 Hz) and β (13–30 Hz) frequency bands. Consistent with the full-range results, no significant clusters were observed within either frequency band (all *P*s > 0.05; see Fig. 5).

### Go/NoGo task results

**Fig. 6:**
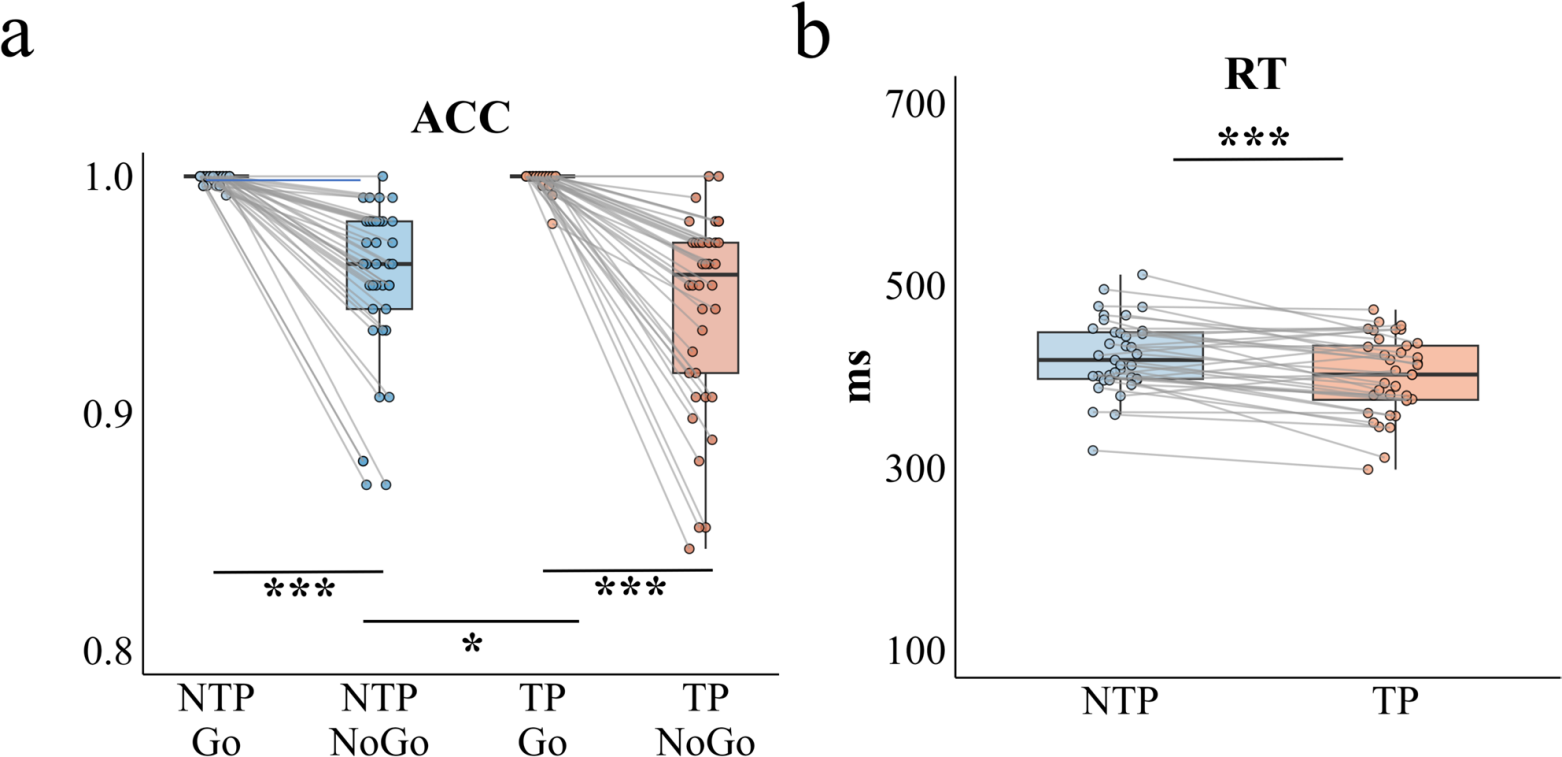
Behavioral performance and average reaction times in the Go/NoGo task under time pressure and no time pressure conditions. **a** Boxplots of average accuracy rates for Go and NoGo trials under time pressure and no time pressure conditions. **b** Boxplots of average reaction times for Go trials under time pressure and no time pressure conditions. Individual data points (dots) represent values for each participant. Gray lines connect paired observations from the same participant across conditions. Asterisks indicate statistical significance: ^*^P < 0.05, ^**^P < 0.01, ^***^P < 0.001

Behavioral Performance: A two-way repeated measures ANOVA was conducted on accuracy rates in the Go/NoGo task, with time pressure and trial type as within-subjects factors. The analysis yielded a statistically significant interaction effect between time pressure and trial type, *F*(1, 39) = 4.471, *P* = 0.041, *η*_p_² = 0.103, indicating differential effects of time pressure across trial types. Follow-up simple effects analyses revealed that time pressure did not significantly affect performance accuracy for either trial type when examined separately. For Go trials, accuracy did not differ between the time pressure condition (M = 0.999, SD = 0.003) and the no time pressure condition (M = 0.998, SD = 0.007), *F*(1, 39) = 0.936, *P* = 0.339, *η*_p_² = 0.023. For NoGo trials, accuracy in the time pressure condition (M = 0.941, SD = 0.048) was also statistically comparable to that in the no time pressure condition (M = 0.951, SD = 0.043), *F*(1, 39) = 3.689, *P* = 0.062, *η*p² = 0.086. In contrast, under no time pressure, participants showed higher accuracy on Go trials (M = 0.998, SD = 0.007) than on NoGo trials (M = 0.951, SD = 0.043), *F*(1, 39) = 46.276, *P* < 0.001, *η*_p_² = 0.543. A similar pattern emerged under time pressure, with Go trials (M = 0.999, SD = 0.003) yielding higher accuracy than NoGo trials (M = 0.941, SD = 0.048), *F*(1, 39) = 56.667, *P* < 0.001, *η*_p_² = 0.592. The analysis revealed no statistically significant difference in accuracy rates between the no time pressure condition (M = 0.975, SD = 0.019) and the time pressure condition (M = 0.970, SD = 0.025), *F*(1, 39) = 2.702, *P* = 0.108, *η*_p_² = 0.065. In contrast, a significant main effect of trial type was observed, demonstrating robust differences in performance between trial conditions, *F*(1, 39) = 59.004, *P* < 0.001, *η*_p_² = 0.602. Specifically, participants showed significantly higher accuracy for Go trials (M = 0.999, SD = 0.006) compared to NoGo trials (M = 0.946, SD = 0.044; see Fig. 6a).

For Go trial RTs, a paired-sample *t*-test showed that RTs were significantly shorter under time pressure (M = 411.440 ms, SD = 57.442 ms) than under no time pressure (M = 432.323 ms, SD = 58.697 ms), *t*(39) = -3.807, *P* < 0.001, 95%CI = (-0.936, -0.261), Cohen’s *d* = 0.602 (see Fig. 6b). 

**Fig. 7:**
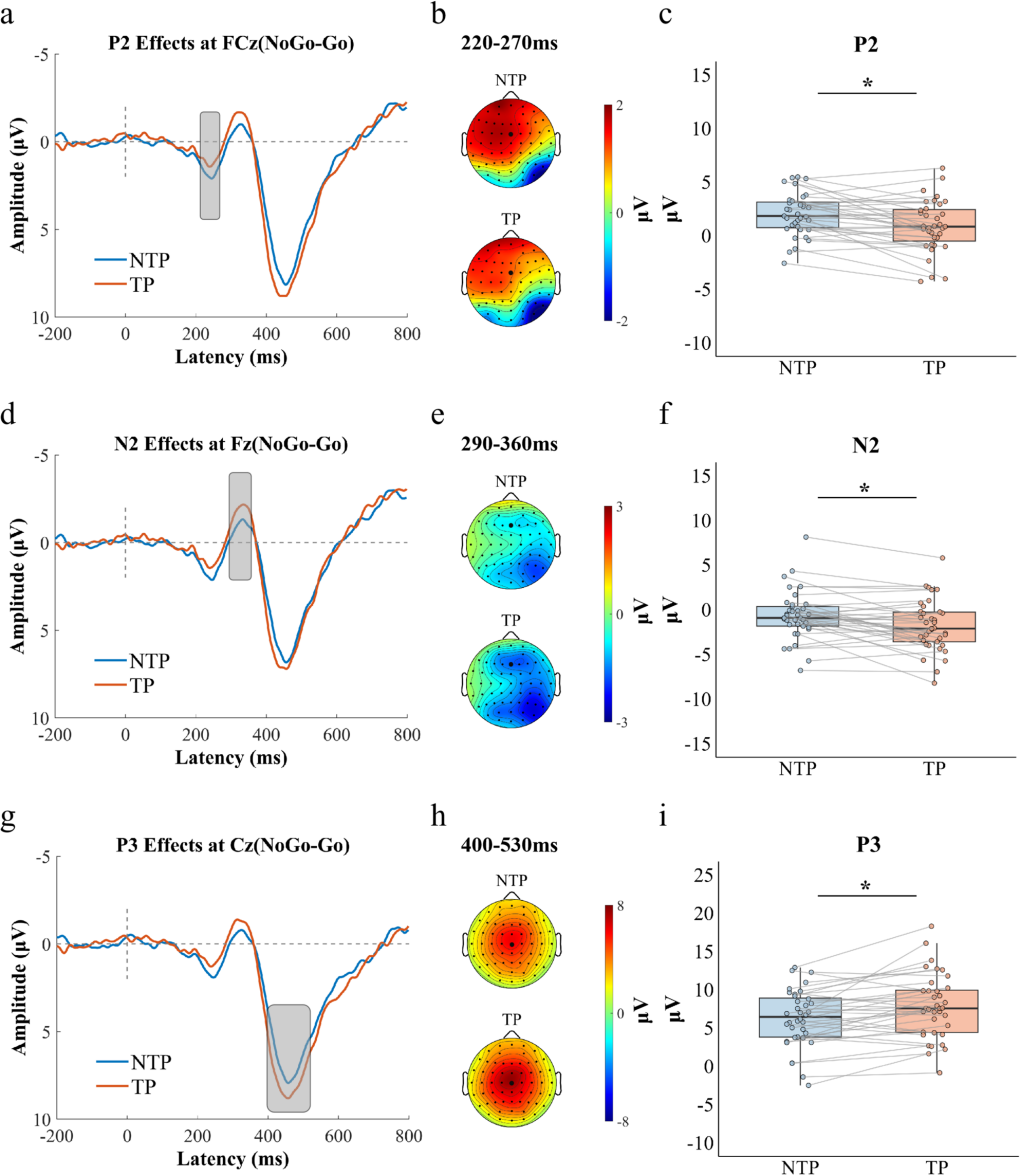
Time pressure effects on P2, N2, and P3 components during response inhibition. **a** Grand-average ERP waveforms at FCz showing NoGo-Go difference waves for no time pressure and time-pressure conditions. **b** The voltage topographies show scalp distributions of the maximum differential P2 (220–270ms). **c** Boxplots of P2 amplitude differences under no time pressure and time pressure conditions. **d** Grand-average ERP waveforms at Fz showing NoGo-Go difference waves for no time pressure and time-pressure conditions. **e** The voltage topographies show scalp distributions of the maximum differential N2 (290–360ms). **f** Boxplots of N2 amplitude differences under no time pressure and time pressure conditions. **g** Grand-average ERP waveforms at Cz showing NoGo-Go difference waves for no time pressure and time-pressure conditions. **h** The voltage topographies show scalp distributions of the maximum differential P3 (400–530ms). **i** Boxplots of P3 amplitude differences under no time pressure and time pressure conditions. Individual data points (dots) represent values for each participant. Gray lines connect paired observations from the same participant across conditions. Asterisks indicate statistical significance: ^*^P < 0.05, ^**^P < 0.01, ^***^P < 0.001

ERP Results: Paired-sample *t*-tests were conducted to compare difference wave amplitudes (P1, N1, P2, N2, and P3) under time pressure and no time pressure conditions. Paired-sample t-tests revealed no significant differences in P1 amplitude at Oz (60–85ms), N1 amplitude at Fz (110–150 ms) between time pressure conditions (all *P*s > 0.05). However, significant effects emerged in later cognitive processing stages: The P2 difference wave amplitude in the 220–270 ms time window at the FCz electrode was significantly smaller under time pressure (M = 1.119 µV, SD = 2.471 µV) than under no time pressure (M = 1.791 µV, SD = 2.052 µV), *t*(39) = -2.095, *P* = 0.043, 95% CI = (-0.648, -0.011), Cohen’s *d* = 0.331. The N2 difference wave amplitude in the 290–360 ms time window at the Fz electrode was significantly more negative under time pressure (M = -1.671 µV, SD = 3.017 µV) than under no time pressure (M = -0.837 µV, SD = 2.963 µV), *t*(39) = 2.264, *P* = 0.029, 95% CI = (-0.676, -0.036), Cohen’s *d* = 0.358. The P3 difference wave amplitude in the 400–530 ms time window at the Cz electrode was significantly greater under time pressure (M = 7.568 µV, SD = 4.464 µV) than under no time pressure (M = 6.464 µV, SD = 4.129 µV), *t*(39) = 2.416, *P* = 0.020, 95% CI = (0.059, 0.701), Cohen’s *d* = 0.382 (see Fig. 7).

Time–Frequency Results: In the full-range time–frequency analysis (3–30 Hz, 0–0.8 s), a significant negative spatiotemporal–frequency cluster was identified at the Fz electrode within the β frequency range (13.92–21.97 Hz) between 378 and 704 ms (cluster-level *P* = 0.042). Within this cluster, β band power was significantly higher under the time pressure condition (M = 0.124 dB, SD = 0.55 dB) compared with the no time pressure condition (M = − 0.226 dB, SD = 0.72 dB).

In the θ band, significant negative clusters were observed at the Fz, FCz, and Cz electrodes. At the Fz electrode, a significant negative cluster was detected between 254 and 408 ms (cluster-level *P* = 0.042). Mean θ band power within this cluster was significantly higher under time pressure (M = 2.877 dB, SD = 1.174 dB) than under no time pressure (M = 2.424 dB, SD = 1.161 dB). At the FCz electrode, a significant negative cluster was identified between 244 and 398 ms (cluster-level *P* = 0.03). Within this cluster, θ band power was significantly higher under time pressure (M = 3.089 dB, SD = 1.189 dB) compared with no time pressure (M = 2.615 dB, SD = 1.208 dB). At the Cz electrode, a significant negative cluster was observed between 234 and 424 ms (cluster-level *P* = 0.02). Mean θ band power in this cluster was also significantly higher under time pressure (M = 2.814 dB, SD = 1.186 dB) than under no time pressure (M = 2.282 dB, SD = 1.271 dB).

**Fig. 8:**
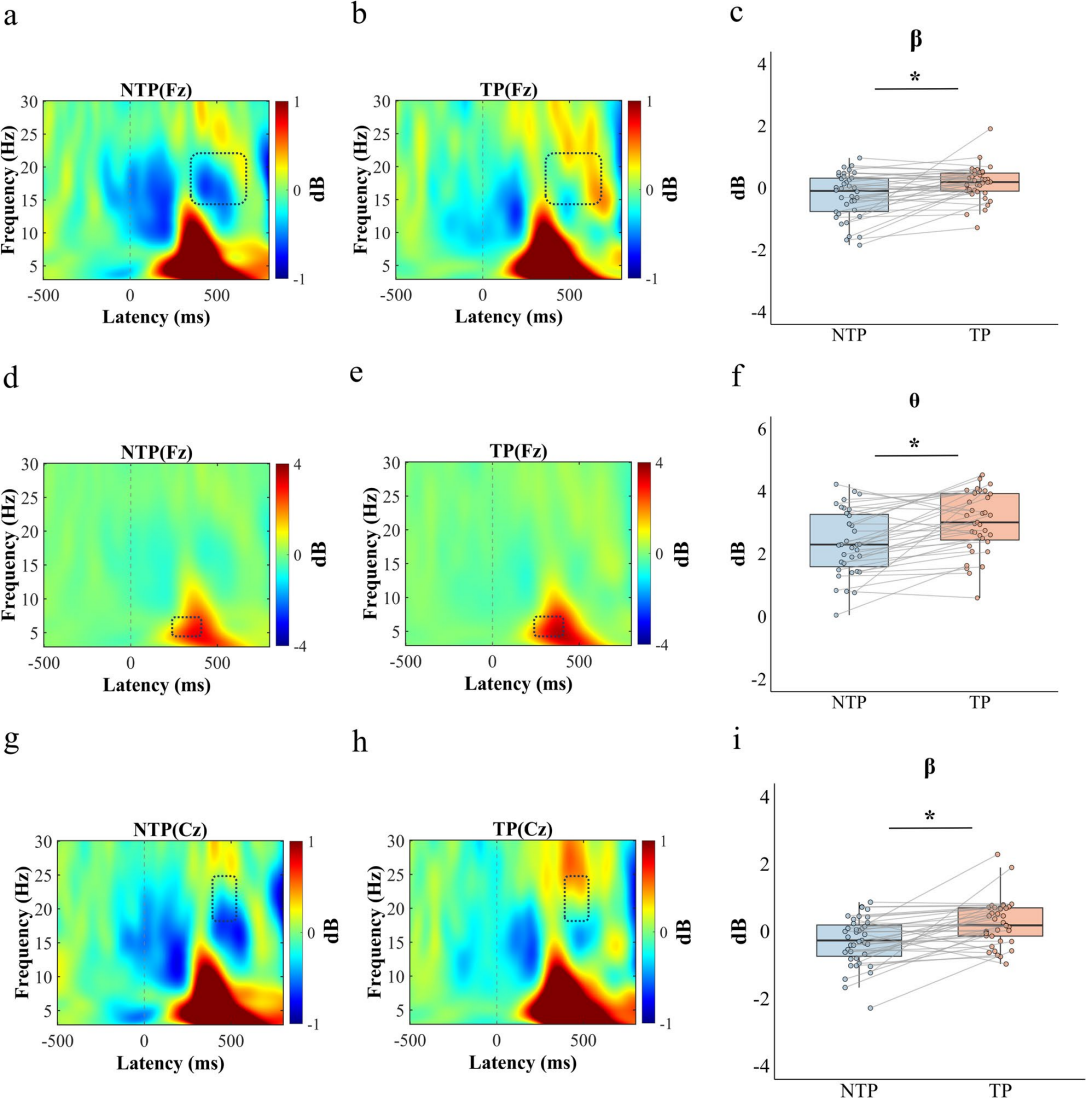
Time-frequency distribution maps under conditions without and with time pressure (NoGo - Go). **a**, **b** Differences in β band power (13.92–21.97 Hz, 378–704 ms) at the Fz electrode site under conditions without and with time pressure. **c** Boxplots of power data for β oscillation under no time pressure and time pressure. These results are based on analyses across the full frequency range. **d**,** e** Differences in θ band power (4.88–7.08 Hz, 254–408 ms) at the Fz (Fz as an exemplar site) electrode site under conditions without and with time pressure. **f** Boxplots of power data for θ oscillation under no time pressure and time pressure. **g**, **h** Differences in β band power (18.07–24.9 Hz, 396–536 ms) at the Cz (Cz as an exemplar site) electrode site under conditions without and with time pressure. **i** Boxplots of power data for β oscillation under no time pressure and time pressure. Individual data points (dots) represent values for each participant. Gray lines connect paired observations from the same participant across conditions. Asterisks indicate statistical significance: ^*^P < 0.05, ^**^P < 0.01, ^***^P < 0.001. Squares outline significant clusters identified by permutation testing, each representing a contiguous region of significant time-frequency modulation

In the β band, significant negative clusters were identified at the Fz, FCz, and Cz electrodes. At the Fz electrode, a significant negative cluster was found within 13.92–21.97 Hz between 378 and 704 ms (cluster-level *P* = 0.03). Within this cluster, β-band power was significantly higher under time pressure (M = 0.124 dB, SD = 0.550 dB) than under no time pressure (M = − 0.226 dB, SD = 0.718 dB). At the FCz electrode, a significant negative cluster was observed within 13.92–22.95 Hz between 386 and 622 ms (cluster-level *P* = 0.04). Mean β-band power within this cluster was significantly higher under time pressure (M = − 0.005 dB, SD = 0.608 dB) compared with no time pressure (M = − 0.341 dB, SD = 0.703 dB). At the Cz electrode, a significant negative cluster was identified within 18.07–24.90 Hz between 396 and 536 ms (cluster-level *P* = 0.04). Within this cluster, β-band power was significantly higher under time pressure (M = 0.160 dB, SD = 0.732 dB) than under no time pressure (M = − 0.269 dB, SD = 0.717 dB; see Fig. 8).

### Correlation analysis results

To examine whether subjective time pressure was associated with changes in behavioral performance or neural activity, two indices of subjective time pressure were used the difference in time-pressure questionnaire scores between the time-pressure and no–time-pressure conditions (ΔTP_questionnaire), and the difference in subjective time-pressure ratings (ΔTP_rating). Both ΔTP_questionnaire and ΔTP_rating were correlated with behavioral measures (Δaccuracy, Δreaction time), ERP component amplitudes (ΔP2, ΔN2, ΔP3), and time–frequency power .

The results revealed that ΔTP_rating was significantly positively correlated with the change in P3 amplitude (ΔP3) in the Flanker task (*r* = 0.357, *P* = 0.024).

## Discussion

The present study investigated the effects of subjective time pressure on inhibitory control, focusing on its behavioral and neural dissociations between interference control (Flanker task) and response inhibition (Go/NoGo task). By integrating behavioral, ERP, and oscillatory analyses, our findings provide a comprehensive understanding of how time pressure modulates distinct inhibitory processes, revealing both shared and unique neural mechanisms underlying these effects.

Across the two inhibitory control tasks, the present study revealed distinct behavioral effects of time pressure, suggesting that temporal constraints do not uniformly impair inhibitory performance but instead exert selective influence depending on task-specific cognitive demands. In the Flanker task, time pressure led to faster overall responses, with RTs significantly reduced under time-pressure conditions. Importantly, this RT facilitation did not differ between congruent and incongruent trials, indicating that participants sped up uniformly rather than selectively adjusting responses to conflict. Nevertheless, accuracy decreased specifically for incongruent trials, and the incongruent–congruent accuracy difference was further enlarged under time pressure, reflecting increased difficulty in resolving conflict when processing time is limited. This pattern is consistent with a speed–accuracy trade-off (SAT), in which participants prioritize speed under temporal constraints, leading to selective reductions in accuracy when task demands are high [62, 63]. In other words, while overall RTs indicate global acceleration under time pressure, the accuracy data reveal that conflict resolution suffers disproportionately.

By contrast, in the Go/NoGo task, overall accuracy for Go and NoGo trials was largely preserved, although the slight increase in the NoGo–Go difference under time pressure suggests that inhibitory responses were somewhat more vulnerable to temporal constraints than simple response execution. These patterns align with diffusion-model accounts of attentional selection [64], which propose that task-specific cognitive demands modulate the extent to which participants trade off speed against accuracy. Taken together, the behavioral results indicate that time pressure selectively affects inhibitory control depending on task structure: it impairs interference control in the Flanker task while exerting a subtler impact on response inhibition in the Go/NoGo task.

The absence of time-pressure effects on the early sensory components P1 and N1 across both inhibitory-control tasks suggests that time pressure does not alter early perceptual encoding or initial attentional orienting [65]. These components reflect exogenous, stimulus-driven processing that typically occurs before cognitive control operations are engaged; thus, their stability across conditions indicates that the influence of time pressure emerges at later, higher-order stages rather than at the level of initial sensory processing. Previous research suggests that reduced P2 amplitudes have been linked to both more efficient filtering of perceptual input—suppressing stimulus features that are not required for response selection and thereby reducing perceptual load [43 44]—and to early stimulus categorization processes [28], in which fewer perceptual resources are needed to distinguish between task-relevant stimulus categories (Go vs. NoGo). Within this framework, the P2 reduction observed under time pressure in the present Go/NoGo task may reflect a shift in early perceptual processing that facilitates rapid categorization of stimuli. Under urgent response demands, participants might prioritize processing of task-relevant categorical information while down-weighting non-essential visual details, such as fine-grained letter features that do not affect the Go/NoGo decision. This interpretation is consistent with prior reports linking P2 amplitude to early perceptual and attentional processes, but should be regarded as a possible explanation rather than a confirmed mechanism. In contrast, this mechanism is unlikely to apply to the Flanker task. The P2 modulation typically observed in Flanker paradigms—larger P2 amplitudes for incongruent compared with congruent trials—reflects selective attentional engagement and the processing of conflict-related stimulus features [66, 67]. No significant differences were observed in the P2 component between the time-pressure and no-time-pressure conditions, which may indicate that early inhibitory mechanisms have limited capacity to selectively suppress distractor stimuli. Flanker stimuli are not entirely irrelevant, but may provide response-related information [68]. Under interference conditions, participants may therefore engage in relatively comprehensive processing of both target and flanker stimuli to resolve conflicts at the stimulus and response-selection levels [43]. In this context, relying solely on early-stage suppression of flanker information may be insufficient to ensure stable response selection.

Moreover, in the Flanker task, time pressure significantly enhanced the amplitudes of the N2 and P3 difference waves (incongruent–congruent). Regarding the functional interpretation of the fronto-central N2, existing evidence links it to both conflict monitoring and early inhibitory demands [69–71]. The enhanced N2 may reflect an overall intensification of conflict processing, which could represent either a passive readout of amplified conflict signals or an active signal of upregulated inhibitory control. The later-appearing P3 difference wave—typically linked to attentional resource allocation and context updating [37, 72]—suggests greater cognitive engagement during conflict resolution. Importantly, the correlation analysis showed that individuals who experienced a larger increase in subjective time pressure (ΔTP) also exhibited larger increases in the P3 difference wave. This association indicates that the subjective experience of being pressured, rather than the experimental manipulation alone, may be a key driver of the enhanced neural recruitment observed under time pressure. In other words, participants who experienced stronger subjective pressure showed either greater engagement of cognitive resources during later-stage inhibitory control, or simply a passive amplification of neural responses due to increased conflict. This pattern suggests that P3 amplitude is modulated by the subjective appraisal of task demands under time constraints, reflecting an increased neural response when task urgency is perceived.

Turning to the Go/NoGo task, time pressure increased both the N2 and P3 difference wave amplitudes (NoGo–Go). Prior research has linked the N2 to conflict monitoring [73, 74] as well as an early premotor inhibition process that suppresses prepotent responding [75]. The enhanced N2 under time pressure may therefore reflect either heightened conflict triggered by the need to override prepotent Go responses, or an increased demand for early inhibitory control in order to prevent premature response activation. The subsequent P3—commonly associated with the implementation of motor inhibition [76–78] and the allocation of cognitive resources for response suppression [79, 80]—was also enlarged under time pressure. This enhancement likely indicates greater engagement of late-stage inhibitory processes or more control-related processing demands. It should be noted that, in line with findings from Go/No-Go probability studies [81] and cue–probe paradigms such as the AX-CPT task [82], some of the observed N2 and P3 enhancements under time pressure may also reflect anticipatory or preparatory processes. Specifically, variations in trial type probability can influence participants’ response expectations and preparatory arousal, which in turn modulate inhibitory-related ERP components.

The present ERP results indicate that the impact of time pressure on inhibitory control follows a hierarchical and task-specific compensatory pattern, although the ultimate neural mobilization fails to translate into efficient behavioral improvement. Specifically: early exogenous perceptual processing (P1/N1) remained stable, suggesting that time pressure does not affect initial sensory encoding. At the intermediate perceptual categorization stage, the modulation of the P2 component revealed a task-dependent adaptive strategy: in the Go/NoGo task, the reduced P2 amplitude may reflect an optimization toward rapid and parsimonious categorical processing of stimuli, whereas in the Flanker task—where conflict detection is indispensable—the P2 remained unaffected. During the core control processing stage, enhancements of both the N2 and P3 may reflect greater engagement of cognitive control resources, but they may also simply represent passive responses to heightened conflict under time pressure. When considered together with the poorer behavioral performance relative to the no-time-pressure condition, these findings may indicate that time pressure impairs inhibitory control.

While ERP analyses revealed the temporal dynamics of inhibitory control under time pressure, the time–frequency results provide important complementary insights into the oscillatory mechanisms underlying these processes. Specifically, our findings demonstrate task-dependent modulations in θ and β band power, suggesting the differential impact of time pressure on interference control and response inhibition at the neural oscillation level.

In the Flanker task, no significant differences were observed across the full frequency range or in the θ and β band power between time pressure and no time pressure conditions across multiple time windows. This suggests that, despite enhanced N2 and P3 amplitudes at the ERP level, the underlying oscillatory dynamics remain relatively stable. One interpretation is that interference control under time pressure relies more heavily on time-locked, phasic neural activity (as reflected in ERPs), rather than sustained oscillatory power changes. Alternatively, the cognitive system may recruit transient, localized resources without broad modulations in frequency-specific power.

In contrast, the Go/NoGo task showed significant increases in θ and β band power under time pressure. The elevation of θ-band power at earlier to intermediate stages of inhibitory processing may be associated with enhanced conflict monitoring and increased cognitive control demands [47, 48], a pattern that is broadly consistent with the enhancement of the N2 component observed in the ERP analysis. Subsequently, the increase in later β band power may be related to the role of β band oscillations in motor inhibition and the suppression of prepotent response tendencies [50–52]. Importantly, these neural modulations were not accompanied by improvements in behavioral performance. Although NoGo accuracy itself did not differ significantly between conditions, the widening NoGo–Go accuracy gap suggests a relative decline in inhibitory control under time pressure. Taken together, the observed increases in θ and β band activity may reflect greater recruitment of cognitive and motor control resources in response to elevated conflict demands, but may also represent a passive neural response to heightened conflict rather than more efficient inhibitory control. In combination with the behavioral findings, these results suggest that time pressure may impair, rather than enhance, inhibitory control.

Notably, these oscillatory changes were not observed in the Flanker task, indicating that response inhibition under time pressure may trigger a more pronounced and sustained oscillatory reconfiguration. Together, these findings support the notion that time pressure differentially affects neural oscillations depending on task demands. In interference control, time pressure appears to modulate event-related potentials without altering the broader spectral profile. In contrast, response inhibition engages both early and late-stage frequency-specific oscillatory mechanisms, particularly in the θ and β bands. These results underscore the utility of combining ERP and time–frequency analyses to more comprehensively understand the neurocognitive impact of temporal stressors on inhibitory control.

### Theoretical implications

This study systematically examined the effects of subjective time pressure on two types of inhibitory control at behavioral, ERP, and time–frequency levels, providing multi-level theoretical insights into the internal structure and neural mechanisms of executive function.

First, the results demonstrate that time pressure does not uniformly affect inhibitory control but exerts dissociable effects on interference control and response inhibition. This pattern aligns with Nigg’s taxonomy of inhibitory processes [15], indicating that external pressure can selectively modulate distinct inhibitory subprocesses in a task-dependent manner.

Second, ERP results revealed that time pressure primarily modulated later cognitive control–related components (N2 and P3), without affecting early perceptual processing (P1 and N1). This suggests that the influence of time pressure emerges at relatively later stages of information processing, rather than during initial sensory encoding, thereby refining theoretical perspectives on how time pressure shapes inhibitory control.Third, task-specific modulations were observed in the time–frequency domain: the Flanker task did not show significant changes in band power, whereas the Go/NoGo task exhibited enhanced θ and β oscillations. These findings suggest that response inhibition relies more heavily on sustained or network-level oscillatory regulation than interference control. The results further clarify the functional differentiation between ERP and time–frequency measures across inhibitory tasks, providing a more fine-grained understanding of the neural mechanisms underlying cognitive control.

Fourth, individual differences in subjective time pressure were correlated with changes in P3 amplitude in the Flanker task, this highlights a new theoretical avenue for investigating the interplay among task demands, subjective experience, and neural resource deployment.

In summary, this study advances our understanding of the structure of inhibitory control, the mechanisms by which time pressure exerts its effects, and the ERP–time–frequency correspondence, offering new empirical evidence for models of executive function.

### Practical implications

The practical implications of this study are fourfold, relating to task design, cognitive assessment, and understanding cognitive strategies under pressure.

First, the findings indicate that subjective time pressure selectively impairs interference control without significantly affecting overall response inhibition. This suggests that in experiments or assessments aimed at measuring interference control, researchers should carefully manage participants’ subjective time perception to avoid confounding effects.

Second, ERP and time–frequency results show that under time pressure, individuals may need to recruit additional cognitive resources to maintain inhibitory function. This insight can guide researchers in designing inhibitory control or cognitive load tasks, helping to identify neural markers of “compensatory control” rather than misinterpreting increased neural activity as enhanced inhibitory capacity.

Third, the sensitivity of θ and β oscillations to response inhibition under pressure highlights these oscillatory measures as potential neural markers for evaluating control mechanisms in stressful conditions. Such information can inform the selection of appropriate neurophysiological indices in future studies involving pressure manipulations.

Fourth, the observed relationship between changes in subjective time pressure and P3 modulation underscores the importance of recording participants’ subjective experiences as covariates in experimental designs. Doing so can improve both experimental validity and the interpretation of behavior–neural relationships.

Overall, this study provides concrete guidance for precisely controlling pressure factors, selecting suitable neural measures, and understanding the differential impact of time pressure on inhibitory processes, thereby supporting more rigorous design, measurement, and interpretation in future research on inhibitory control.

### Limitations and future directions

Although this study revealed the task-specific effects of time pressure on inhibitory control and provided rich neural and behavioral evidence, several limitations remain and should be addressed in future research.

First, although the time pressure manipulation effectively induced a subjective sense of urgency, the experimental setting may not fully capture the complexity of real-world time-constrained situations, which often involve emotional arousal, multitasking, or environmental uncertainty. Future studies could adopt more ecologically valid paradigms—such as virtual reality tasks or dynamic decision-making scenarios—to better simulate realistic stressors.

Additionally, the absence of significant time–frequency effects in the Flanker task does not imply a lack of involvement of neural oscillations in interference control. Rather, it may reflect the inherently time-locked nature of this inhibitory process, which is more readily captured by ERP components. This suggests that oscillatory changes could be more subtle or spatially distributed, necessitating more refined analytic methods—such as single-trial modeling or functional connectivity analyses—to detect them. Future research could also investigate whether increasing cognitive load or manipulating task parameters to enhance interference saliency might reveal clearer oscillatory signatures.

Third, the current sample consisted of healthy young adults, which limits the generalizability of the findings. Given that stress responses and inhibitory capacity vary across age groups and clinical populations (e.g., older adults, individuals with anxiety, or those with attention deficit disorders), future research should further explore how time pressure affects different populations and what factors may modulate this effect.

Fourth, a further limitation of the present study concerns the conceptual overlap between interference control and response inhibition. Although the flanker task is commonly used to index interference control and the Go/NoGo task to index response inhibition, these two forms of inhibitory control are not entirely independent and likely rely on partially shared cognitive and neural mechanisms. Consequently, both tasks may engage overlapping processes such as conflict monitoring and top–down control, in addition to their dominant inhibitory demands. Therefore, the observed differences between task conditions should be interpreted as reflecting relative differences in dominant control demands rather than strictly dissociable inhibitory subprocesses. Future studies employing tasks or analytical approaches that allow a more fine-grained separation of inhibitory components would be valuable to further clarify the specificity of these effects.

## Conclusion

This study suggests that subjective time pressure may exert selective and task-dependent effects on inhibitory control, with different patterns observed in the neurodynamics of interference control and response inhibition. Although time pressure was associated with generally faster responses, it was linked to reduced conflict resolution accuracy in the flanker task while having relatively limited effects on performance in the Go/NoGo task, pointing to differential vulnerability across inhibitory subprocesses.

At the neural level, early sensory encoding indexed by P1 and N1 appeared to remain relatively stable across tasks, suggesting that initial sensory processing may not be substantially affected by time constraints. In contrast, effects were mainly observed at mid- to late-stage processing. Task-related modulation of the P2 may reflect adaptive adjustments in early perceptual categorization, whereas enhancements of N2 and P3 amplitudes in both tasks could indicate either increased recruitment of cognitive control resources or a passive amplification of neural responses under heightened demands.

Time–frequency analyses further indicated that these neural modulations were accompanied by increases in θ and β band activity during response inhibition, whereas no comparable oscillatory changes were detected during interference control. This pattern may point to fundamental differences in how these two inhibitory systems respond to temporal constraints.

Importantly, despite these neural modulations, behavioral performance did not show corresponding improvements under time pressure, suggesting that the enhanced neural activity is more likely to reflect increased processing demands or compensatory engagement rather than more efficient inhibitory control. Taken together, these findings highlight the complex, multi-level nature of inhibitory control under time pressure and underscore the value of integrating behavioral, ERP, and oscillatory measures to capture its nuanced neurocognitive dynamics.

## Supplementary Information

Below is the link to the electronic supplementary material.

Supplementary Material

## Data Availability

The data underlying this article will be shared on reasonable request to the corresponding author.
