# Supplementary material for: How time pressure disrupts inhibitory control: neural dissociations between interference and response inhibition
Source: Behav Brain Funct. 2026 Apr 16;22:23. doi: 10.1186/s12993-026-00331-3 (PMC13202991; doi:10.1186/s12993-026-00331-3)
Supplement: Supplementary file 1 — Supplementary Material [file 12993_2026_331_MOESM1_ESM.docx]

|  | subjective time-pressure ratings | | |
| --- | --- | --- | --- |
|  | *r* | *P* | 95% CI |
| Flanker_ACC | -0.228 | 0.156 | [-0.504 0.090] |
| Flanker_RT | 0.131 | 0.419 | [-0.188 0.426] |
| Flanker_N2 | -0.047 | 0.774 | [-0.353 0.269] |
| Flanker_P3 | 0.357 | 0.024 | [0.051 0.602] |
| Go/NoGo_ACC | -0.003 | 0.984 | [-0.315 0.309] |
| Go/NoGo_RT | 0.197 | 0.223 | [-0.122 0.479] |
| Go/NoGo_N2 | -0.052 | 0.750 | [-0.358 0.264] |
| Go/NoGo_P2 | -0.131 | 0.420 | [-0.425 0.188] |
| Go/NoGo_P3 | -0.166 | 0.305 | [-0.454 0.153] |
| Go/NoGo(3-30Hz)_Fz | -0.241 | 0.135 | [-0.514 0.077] |
| Go/NoGo(4-7Hz)_FCz | -0.253 | 0.115 | [-0.523 0.064] |
| Go/NoGo(4-7Hz)_FCz | -0.225 | 0.162 | [-0.502 0.093] |
| Go/NoGo(4-7Hz)_Cz | -0.261 | 0.104 | [-0.529 0.055] |
| Go/NoGo(13-30Hz)_Fz | -0.241 | 0.135 | [-0.514 0.077] |
| Go/NoGo(13-30Hz)_FCz | -0.089 | 0.585 | [-0.390 0.229] |
| Go/NoGo(13-30Hz)_Cz | -0.098 | 0.549 | [-0.397 0.221] |

All variables in the table represent the difference between the time-pressure (TP) and no-time-pressure (NTP) conditions (TP–NTP). The table shows the correlations between ΔTP_rating and the significant Δ measures in the Flanker/Go–NoGo task, including correlation coefficients (r), p-values, and 95% confidence intervals (CI).
